# The protective role of parent-child communication: how disclosure moderates the link between risk profiles and adolescent sexting behaviors

**DOI:** 10.3389/fpsyg.2026.1790264

**Published:** 2026-03-18

**Authors:** Michal Dolev-Cohen, Hila Shaul

**Affiliations:** Educational Counseling Program, Oranim Academic College, Kiryat Tiv'on, Israel

**Keywords:** adolescents, disclosure to parents, harm minimization, sexters’ profiles, victim blaming

## Abstract

**Introduction:**

Adolescent sexting is a developmentally embedded behavior reflecting adolescents’ negotiation of autonomy, intimacy, and social norms within digital contexts. While prior research has demonstrated heterogeneity in adolescents’ sexting involvement, less is known about how family relational processes influence the translation of risk related attitudes into behavior. Guided by social control theory and differential association theory, this study examined whether adolescents’ disclosure to parents moderates the relationship between attitudinal profiles and sexting behaviors.

**Methods:**

Data were collected from 345 Israeli adolescents and young adults who retrospectively reported on their experiences during high school. Latent profile analysis was conducted to identify groups of adolescents based on sexting norms, harm minimization, victim blaming, and social desirability. Subsequent moderation analyses examined whether adolescents’ disclosure to parents moderated the association between these profiles and engagement in active, passive, and coercive sexting behaviors.

**Results:**

Three distinct profiles emerged: non sexters, advocates of sexting norms, and harmful sexters. Adolescents’ disclosure to parents demonstrated a consistent protective effect. When disclosure levels were high, differences between profiles in active, passive, and coercive sexting behaviors were not statistically significant. In contrast, among adolescents reporting low disclosure to parents, those in the harmful sexters profile reported significantly higher involvement in all forms of sexting compared to adolescents in the other profiles.

**Discussion:**

The findings highlight adolescent disclosure to parents as a central developmental mechanism that regulates the behavioral expression of risk related norms and attitudes. By situating sexting within parent child communication dynamics, the study underscores the importance of relational processes in supporting adaptive development and identifies open disclosure as a key target for developmentally informed prevention and intervention efforts.

## 1 Introduction

### 1.1 Sexting behaviors and normative risk frameworks

Adolescent sexting has been widely discussed in the literature as a behavior that oscillates between normative sexual exploration and potential digital harm. Rather than revisiting this debate, the present study examines how adolescents’ positions within distinct attitudinal and normative profiles shape their engagement in different forms of sexting, and how parental disclosure may alter these associations.

Sexting, defined as the digital exchange of sexually explicit texts or images, is increasingly common among adolescents (Dir et al., 2013; Klettke et al., 2019; Mori et al., 2022). This practice often takes place within romantic relationships or intimate interactions, leading many researchers to conceptualize consensual sexting as part of normative adolescent sexual development (Pistoni et al., 2023; Maes and Vandenbosch, 2022). Within consensual contexts, sexting may serve as a means of expressing affection, sexual curiosity, and intimacy (Cooper et al., 2016; Döring, 2014). At the same time, sexting remains controversial due to its potential risks, particularly in situations where consent is absent or ambiguous (Dolev-Cohen et al., 2023; Döring, 2014; Livingstone and Mason, 2015). The literature commonly distinguishes between active sexting, which involves the creation and distribution of sexual content, and passive sexting, which refers to receiving sexts or sext requests (Molla-Esparza et al., 2023). Non-consensual sexting, including coercion, unwanted dissemination of images, or unsolicited explicit content, has been consistently linked to psychological distress, victimization, and adverse social outcomes (Doyle et al., 2021; Lu et al., 2021). To address this aim, the present study integrates four conceptual domains: adolescents’ sexting behaviors, normative and attitudinal risk frameworks (including victim blaming and harm minimization), person-centered profiling approaches, and parent–child communication. Together, these domains provide the basis for examining parental disclosure as a central moderating mechanism linking adolescents’ attitudinal profiles to their sexting behaviors.

### 1.2 Person-centered approaches to adolescent sexting

From a social-developmental perspective, sexting may function as a mechanism through which adolescents negotiate peer belonging and social visibility. In such contexts, apparent consent may coexist with implicit social pressures, complicating adolescents’ interpretations of agency and choice (Giletta et al., 2021; Van Ouytsel et al., 2017; Ringrose et al., 2021).

Beyond individual behavior, societal and cultural processes play a central role in shaping responses to unwanted or coercive sexting. Both individuals and social institutions may downplay the severity of sexual harm, blur moral boundaries, and attribute responsibility to victims (Dolev-Cohen and Henry, 2025). These perceptions are influenced by cultural, gender, and social contexts (Henry et al., 2020; Powell et al., 2019). Victim blaming is often manifested through assigning responsibility seen as lying with the victim, particularly when adolescents initially shared intimate content that was later distributed without consent (Cricenti et al., 2022). Social norms may further legitimize the non-consensual distribution of intimate images by framing such harm as marginal or inevitable within digital environments (Henry et al., 2020). Importantly, attitudinal constructs such as victim blaming and harm minimization do not operate in isolation. Their translation into actual behavior is likely shaped by adolescents’ relational contexts, particularly the quality of communication with parents. From a developmental and social control perspective, normative beliefs may predict sexting behavior only to the extent that they are not counterbalanced by open parent–child communication. Thus, parental disclosure should be conceptualized not merely as a general protective factor, but as a relational mechanism that may regulate whether risk-related attitudes are enacted behaviorally.

To identify adolescents’ engagement in risky sexting behaviors that may lead to sexual harm, prior research has increasingly relied on person-centered approaches, including latent profile analysis. Studies conducted in Israel have distinguished between non-sexters and sexters, showing that adolescents who engage in sexting report lower family and peer cohesion and stronger endorsement of peers’ sexting norms, and are more likely to be secular and not involved in a romantic relationship (Dolev-Cohen, 2024). Research conducted in the United States identified four distinct profiles of adolescents differing in sexting involvement and sexual behavior. The largest group was characterized by low engagement in both sexting and sexual activity, whereas other profiles reflected varying combinations of sexting, sexual activity, and risk behaviors (Mori et al., 2021). Additional findings suggest that adolescents who send unsolicited sexts exhibit greater emotional instability compared to consensual sexters and adolescents not involved in sexting (Kokkinos et al., 2024). Across studies, individual and contextual risk factors, including impulsivity, sensation seeking, and peer influence, have been consistently linked to sexting involvement (Pistoni et al., 2023; Maheux et al., 2020). Although person-centered approaches have successfully captured heterogeneity in adolescents’ sexting involvement, most studies have focused primarily on behavioral patterns, personality traits, or peer-related influences. Family relational processes have rarely been incorporated into latent profile frameworks, and even less frequently examined as moderators of profile–behavior associations. Consequently, it remains unclear whether attitudinal risk configurations exert uniform behavioral effects across relational contexts, or whether their expression depends on the quality of parent–child communication. The present study addresses this gap by integrating adolescent disclosure to parents into a person-centered design as a relational mechanism that conditions the behavioral implications of distinct sexting-related profiles.

### 1.3 Parent-child communication about sexting and disclosure

Parents play a pivotal role in adolescents’ online sexual behavior. Protective factors such as parental involvement, open family communication, and adolescents’ self-regulation capacities are consistently associated with lower engagement in risky sexting (Bianchi et al., 2021; Corcoran et al., 2022).

Parents have been shown to play a crucial role in mediating adolescents’ sexting behaviors and reducing associated risks (Dolev-Cohen and Ricon, 2022; Vanwesenbeeck et al., 2018). For girls, lower levels of family communication were associated with increased engagement in various forms of sexting, including those with potential risks (Bianchi et al., 2021). Studies on boys indicated that the presence of parental rules regarding sexting, such as restrictions on sending explicit messages, was associated with a decrease in such behaviors (West et al., 2014). Later findings confirmed this association, showing that clearly defined parental rules about digital content consumption contributed to adolescents’ negative attitudes toward sexting and a reduction in their engagement in it (Confalonieri et al., 2020).

Additionally, it was found that different types of parental mediation produce different outcomes in relation to the adolescents’ motivations for engaging in sexting. Restrictive technological mediation may lead to increased sexting by adolescents who engage in it out of developmental sexual motivation. Therefore, it is recommended to promote open dialogue and encourage healthy attitudes toward sexuality and sexting (Dolev-Cohen, 2023). Overall, fostering open communication and providing consistent parental support have been found to create a safer environment for the sexual development of adolescents, including their experiences with sexting (Pistoni et al., 2023). In sum, despite its prevalence, sexting by adolescents may indicate risk, especially when performed unwillingly or under social pressure. At the same time, it was found that parents play a significant role in reducing risks, with open communication and mediation helping reduce involvement in risky sexting. Thus, the present study sought to examine how adolescents’ willingness to share experiences with their parents serves as a protective factor in various profiles of adolescent sexters.

Taken together, the literature suggests that adolescents’ sexting behaviors emerge at the intersection of normative beliefs, peer environments, and family relational dynamics. However, these domains have typically been examined in parallel rather than in an integrated model. While attitudinal and normative frameworks explain variability in adolescents’ endorsement of sexting-related beliefs, and person-centered approaches identify distinct risk configurations, the moderating role of parental disclosure in shaping whether such configurations translate into behavior remains underexplored. Grounded in social control theory and differential association theory, the present study advances an integrative framework in which adolescent disclosure to parents functions as a relational regulator of profile-specific risk. In this framework, social control theory provides the foundation for conceptualizing disclosure as an informal regulatory bond to parental norms, whereas differential association theory illuminates how harmful sexting profiles may emerge within peer contexts that normalize exploitative behaviors.

Accordingly, the present study pursued two primary aims: (1) to identify latent profiles of adolescents based on sexting-related norms, harm minimization, victim blaming, and social desirability; and (2) to examine whether adolescents’ disclosure to parents moderates the association between these profiles and active, passive, and coercive sexting behaviors.

## 2 Methods

### 2.1 Participants and procedure

The data analyzed in the present study were drawn from a broader research project. While based on the same dataset as previous publications, the current analyses address distinct research questions and employ different analytical strategies.

Data were collected during 2025 from a diverse cohort of 345 Israeli youths (44% male). Participants ranged in age from 16 to 26 years (M = 17.78, SD = 1.57), completed a battery of six validated Hebrew instruments via an anonymous digital platform. Participants were recruited through parental outreach. Parents received an invitation and forwarded the survey link to their adolescent children and all minor participants provided parental consent before completing the questionnaires. Outreach to young adults (18–21 years) was conducted via postings in relevant young adult groups. Of the total sample, 169 adolescents (51.7%) reported engagement in sexting behaviors (Table 1).

**Table 1:** Background characteristics of the participants (*N* = 345).

Characteristic	*N* = 345^1^
Gender	
Male	153 (45%)
Female	190 (55%)
Unknown	2
Age	17.78 (1.57; 15.00–26.00)
Religiosity	
Secular	215 (62%)
Religious	114 (33%)
Traditional	16 (4.6%)

^1^*n* (%); ^2^Mean (SD; range).

The study was approved by the institutional Ethics Review Board.

Although the sample includes participants aged 16–26, the present study focuses conceptually on adolescence. All participants were asked about behaviors, attitudes, and disclosure patterns during their high school years. Thus, responses of young adults reflect adolescent experiences rather than current developmental functioning. This approach aligns with prior research relying on retrospective reports to examine adolescent digital behaviors and family processes.

The sample was a convenience sample and was not intended to be nationally representative. Adolescents were recruited through parental outreach, and young adults through online postings. The study aims to identify relational and attitudinal patterns rather than population prevalence estimates.

### 2.2 Measures

#### 2.2.1 Demographics questionnaire

A demographics questionnaire, developed specifically for this study, collected information about the participants, including their age, grade level, gender, and self-identified level of religiosity (non-religious, traditional, and religious).

#### 2.2.2 Social desirability scale

To control for response bias, we administered the Hebrew adaptation (Ben-Zur and Reshef-Kfir, 2003) of Crowne and Marlowe’s (1960) Social Desirability Scale, utilizing the validated eight-item short form. The abbreviated version comprises eight items assessing attitudes, opinions, and personality traits, with participants indicating whether each statement regarding themselves is true or false. A sample item is: “I’m always polite, even with unpleasant people.” Higher scores reflect greater social desirability. In the present study, the internal consistency was relatively low (*ω* = 0.65).

#### 2.2.3 Perception of sexting norms of friends (Dolev-Cohen, 2023)

Adolescents’ beliefs regarding the social acceptability of digital sexual exchange among high school peers and parents were measured using a six-item scale (Dolev-Cohen, 2023). The original version includes six items: three pertaining to perceived peer norms and three to parental norms. For the present study, only the peer-related items were administered. A sample question: “I think that most of my friends send sexts to others”. Responses were recorded on a 5-point Likert scale ranging from 1 (Completely disagree) to 5 (Strongly agree). Internal consistency of the three peer norm items in the current sample was high (ω = 1.00).

#### 2.2.4 Involvement in sexting behavior: image-based sexual abuse – IBSA (Powell et al., 2019)

The original questionnaire comprises two subscales, one assessing online dating behaviors and the other sexual self-image behaviors. For the present study, only the latter subscale was used, which includes 11 items measuring the adolescents’ involvement in sexting: The scale categorized into three subscales: active sexting (six items; e.g., “You sent someone you just met a nude photo, a sexual photo, or a sexual video to flirt with them”), coercive sexting (two items; e.g., “You felt pressured to send a photo or video when you really didn’t want to”), and passive sexting (three items; e.g., “You received an unsolicited nude or sexual photo or video of someone else (not including spam)”). Responses were rated on a 5-point Likert scale ranging from 1 (Never) to 5 (Often). Based on the responses, a dichotomous variable indicating “sexting involvement” was created. Internal consistency for the current sample was high: active sexting (*ω* = 0.95), coercive sexting (ω = 0.74), passive sexting (ω = 1.00).

#### 2.2.5 The sexual image-based abuse myth acceptance - SIAMA (Powell et al., 2019)

The questionnaire consists of 18 items, divided into two subscales. The harm minimization subscale includes 12 items, such as: “It is flattering to women if an ex-partner shares their nude photo with a few close friends.” The victim-blaming subscale includes six items, for example: “A person who sends a nude photo or sexual image to someone else is at least partially responsible if the image ends up on the Internet.” Participants rated each item on a 7-point Likert scale ranging from 1 (Strongly disagree) to 7 (Strongly agree). Higher mean scores reflect stronger tendencies to downplay harm and blame victims of image-based sexual abuse (IBSA). In the present study, internal consistency was ω = 0.88 for the harm minimization subscale and ω = 0.90 for the victim-blaming subscale.

#### 2.2.6 Adolescents’ disclosure to parents (Sher-Censor et al., 2021)

Information management and the transparency of communication between the adolescent and their parents were evaluated using an instrument based on the work of Kerr and Stattin (2000). For the present study, a modified version by Sher-Censor et al. (2021) was used. This version includes 13 items that evaluate adolescents’ disclosure about daily activities occurring outside of parental supervision, such as during school hours or time spent away from home. The scale includes four concealment items (e.g., “If other boys or girls pressure me to do bad things, I won’t share this matter with my parents”) and six disclosure items (e.g., “If I get caught in any illegal behavior, I will turn to my parents to ask for help”). To adapt the instrument to the present study, three additional items were included about online image distribution, for example, “If images of me are distributed online, I will try to hide the matter from my parents.” All items were rated on a 5-point Likert scale ranging from 1 (Not true at all) to 5 (Very true). Internal consistency for the full scale was good (*ω* = 0.85).

### 2.3 Analyses

Missing data were permitted, resulting in 0.81% of the data being absent, with 7 distinct patterns. Jamshidian and Jalal’s non-parametric Missing Completely at Random (MCAR) test indicated that the data were missing completely at random (*Hawkins’s χ^2^*_(4)median_ = 147.69, *p*_median_ = 6.38^−31^, *Anderson-Darling T*_median_ = 0.39, *p* = 0.561). Therefore, we addressed missing data by multiple imputation (MI) (Rubin, 2009) with 10 complete sets, using the *mice* and *micemd* R packages. The reported results represent the pooled outcome of the MI procedure. Before the primary analyses, we also evaluated the normal distribution of all quantitative study measures, conducting a series of Anderson-Darling normality tests. We also assessed the presence of multivariate outliers using the minimum covariance determinant approach with the *robustbase* R package. We found that all measures significantly deviated from normality (all *A* > 1.59, all *p* < 0.0004 or lower). Because 104 observations were identified as multivariate outliers, we used non-parametric and robust statistics to test the study hypotheses.

**Figure 1:**
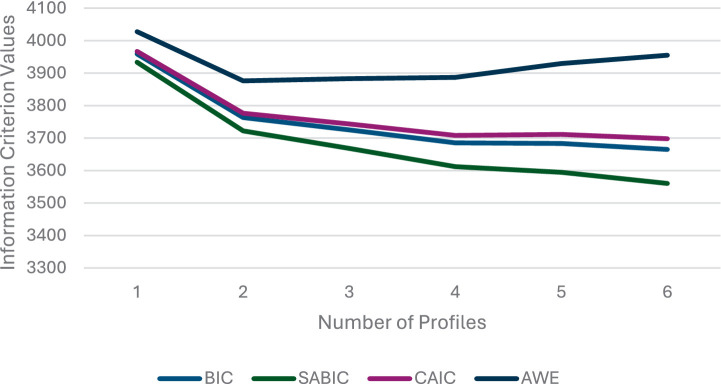
Scree plot of the Bayesian information criterion (BIC), sample-size adjusted Bayesian information criterion (SABIC), consistent Akaike information criterion (CAIC), and approximate weight of evidence criterion (AWE). The “elbow” above the 3-profile solution indicates its superiority.

We began by applying latent profile analysis to estimate distinct latent profiles based on participants’ social desirability, sexting norms, and tendencies toward harm minimization and victim blaming in the case of IBSA behaviors. To this end, we followed the guidelines of Nylund-Gibson and Choi (2018) using the *tidyLPA* R package with MPlus 8.8 (Muthén and Muthén, 2019) structural equation modeling (SEM) integration. We examined one to six possible profiles using unconditional LPA. To decide on the number of profiles, we used the following information (Table 2): (a) information criteria (IC), including the Bayesian information criterion (BIC), sample-size adjusted bayesian information criterion (SABIC), consistent Akaike information criterion (CAIC), and approximate weight of evidence criterion (AWE) approximate fit indices, where lower values indicate superior fit. The ICs were plotted (Figure 1) to identify “elbows” of point of “diminishing returns” in model fit (equivalent to a scree plot in factor analysis); (b) We also used the bootstrapped likelihood ratio test (BLRT), which provides *p*-values to assess whether adding a class leads to a statistically significant improvement in model fit. The BLRT is one of the most robust methods among various modeling conditions (Nylund et al., 2007). (c) We considered the Bayes factor (BF) indices for pairwise comparisons of fit between two adjacent class models, with values greater than 10 suggesting “strong” support for the more complex model. Additionally, we assessed the correct model probability (cmP), which estimates the likelihood of each model being “correct” among all models evaluated. We also assessed how the selected models relate to each other (e.g., are theoretically different).

**Table 2:** Fit indices for the LPAs.

	1 profile	2 profiles	3 profiles	4 profiles	5 profiles	6 profiles
BIC	3959.01	3763.20	3725.36	3685.07	3683.23	**3664.94**
SABIC	3933.63	3721.96	3668.26	3612.11	3594.40	**3560.25**
CAIC	3967.01	3776.20	3743.36	3708.07	3711.23	**3697.94**
AWE	4027.76	3876.18	3882.86	3886.77	3929.36	**3955.28**
BLRT		225.03	67.06	69.51	27.14	**29.02**
BF		17866.81	**16.63**	7.50	1.10	2.50
cmP	0.00	0.00	0.03	0.20	0.22	**0.55**
Entropy		0.99	0.84	0.85	0.74	0.75
LL	−1956.13	−1843.62	−1810.09	−1775.33	−1759.80	−1736.05
% smallest *n*		4.05	4.05	1.15	1.15	1.15

Lower values indicate better model fit. Note. Bold values indicate the best-fitting model according to each fit index. For BIC, SABIC, CAIC, and AWE, lower values indicate better model fit. For BLRT and the Bayes Factor (BF), higher values indicate stronger support for the model compared with the model with one fewer profile. For the cmP (correct model probability), higher values indicate greater probability that the model is the correct one among the tested solutions.

After determining the optimal number of profiles, we used these in moderation analyses to examine whether adolescents’ tendencies to disclose information to their parents moderated the effects of the profiles on sexting behaviors, based on social desirability, sexting norms, and tendencies toward harm minimization and victim blaming. To this end, we conducted a series of multiple regressions with robust standard errors. In these models, sexting behaviors (active, passive, and coercive) served as the outcome variables; profiles (effect coded as −0.5 and 0.5) were included as predictors; children’s disclosure to parents was entered as the moderator; and gender and religiosity served as covariates to adjust for their contribution. Disclosure scores were mean-centered to reduce multicollinearity and to facilitate interpretation of interaction effects. Robust standard errors were calculated using the *sandwich* R package. Significant interactions were further examined through simple slope analyses conducted using the *interactions* R package.

## 3 Results

### 3.1 Latent profile analysis

The results are summarized in Table 2. Fit indices did not converge on a single solution, which is generally the rule rather than the exception in practice (Nylund-Gibson and Choi, 2018). The ICs scree plot (Figure 1) reveals an “elbow” above the three-profile solution, suggesting its superiority, as this inflection point marks a notable reduction in improvements to model fit beyond three profiles. Additionally, the BF score favored the three-profile solution. By contrast, the cmP score and the BLRT test supported a six-profile solution, which, however, included a group comprising only four participants (1.5% of the sample). After considering all fit indices and the negligible sample size of the additional profile in the six-profile solution, we selected the three-profile solution as the optimal model. An entropy score of 84% indicates well-separated profiles (Nagin and Nagin, 2005).

### 3.2 Profile characteristics

**Figure 2:**
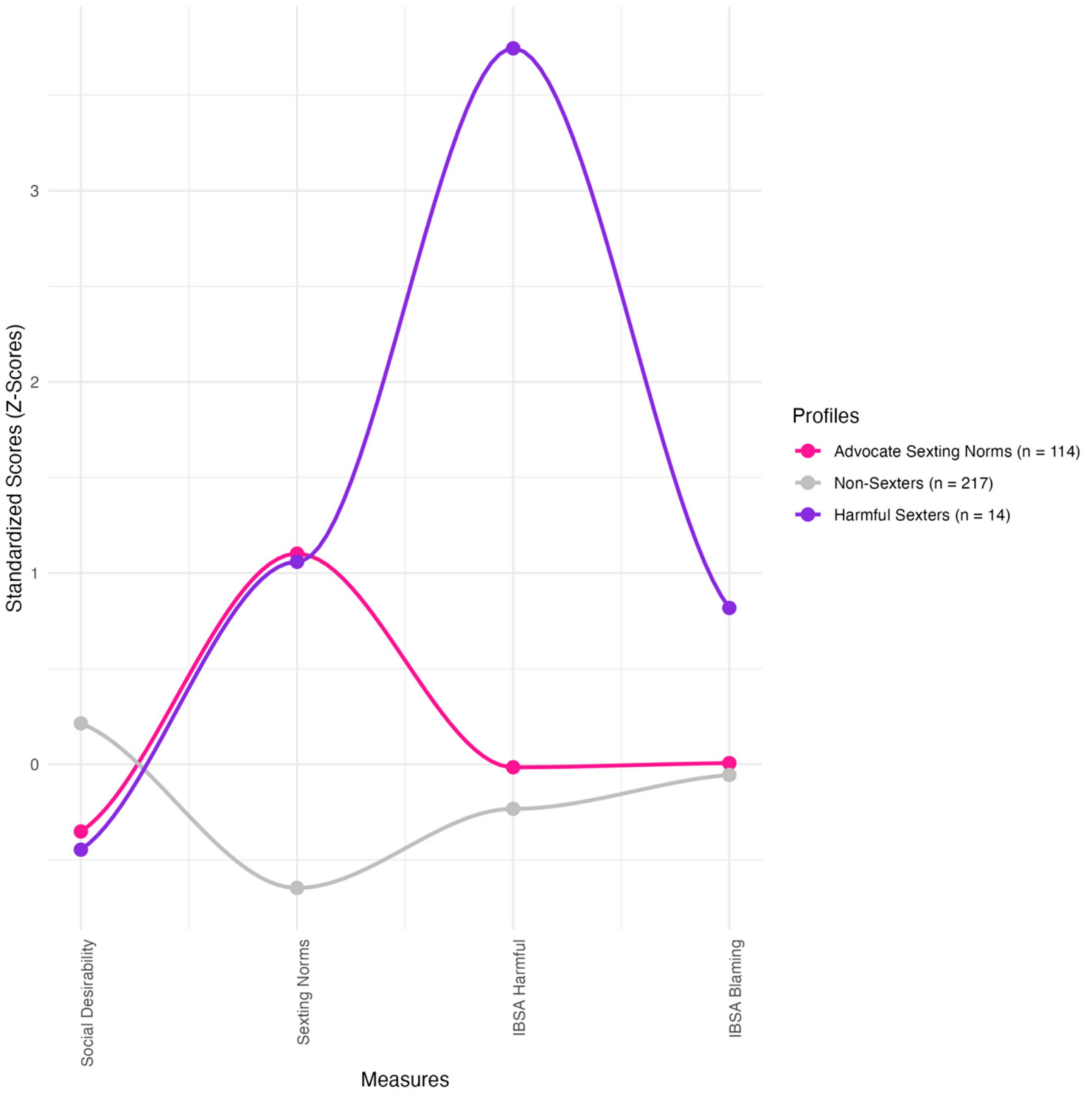
The selected 3-profile solution. The measures used in the LPA are presented on the *x*-axis. Relative scores are presented on the *y*-axis.

The profiles (Figure 2) include the following groups: “non-sexters” (*n* = 217; 62.90%), “advocates of sexting norms” (*n* = 114; 33.04%), and “harmful sexters” (*n* = 14; 4.06%). Non-sexters had below-average scores on all sexting-related measures and a slightly above-average score on social desirability. The non-sexters profile was characterized by weaker sexting-related norms and lower levels of harm minimization and victim blaming in cases of IBSA.

The profile of advocates of sexting norms was characterized by average levels of victim blaming and harm minimization, alongside a moderately elevated acceptance of sexting norms (*z* ≈ 1). They also showed a below-average score on social desirability (*z* = −0.35), indicating lower endorsement of socially desirable responding. Harmful sexters included individuals with exceptionally high scores in harm minimization tendencies (*z* = 3.75), elevated scores in victim blaming (*z* = 0.82), and a heightened inclination toward accepting sexting norms (*z* ~ 1). Finally, the profile was characterized by a below-average score on social desirability (*z* = −0.45).

### 3.3 The moderating effect of the adolescents’ disclosure of information to parents on the association between profiles and sexting behaviors

**Figure 3:**
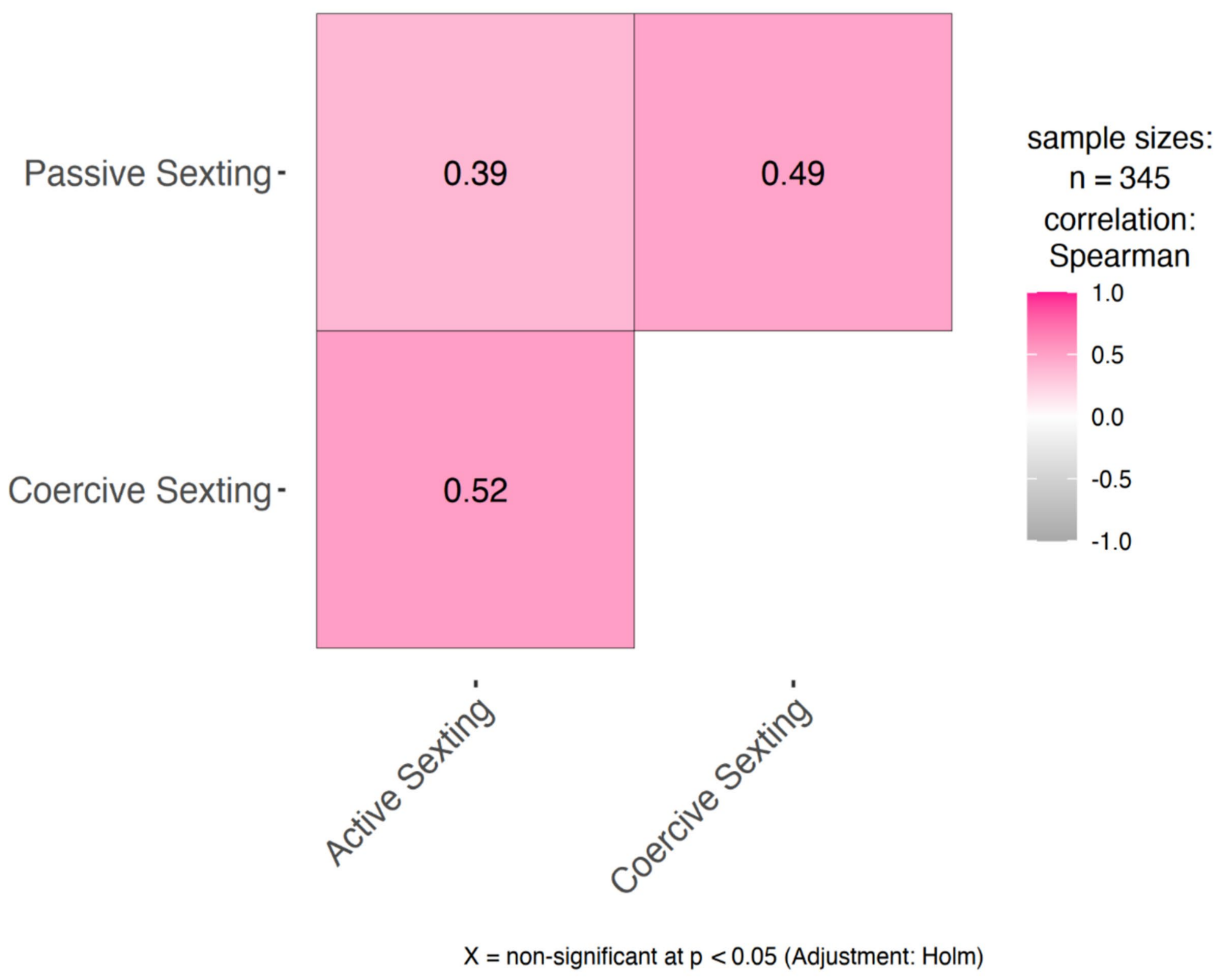
Spearman rho correlations between the sexting behaviors.

**Figure 4:**
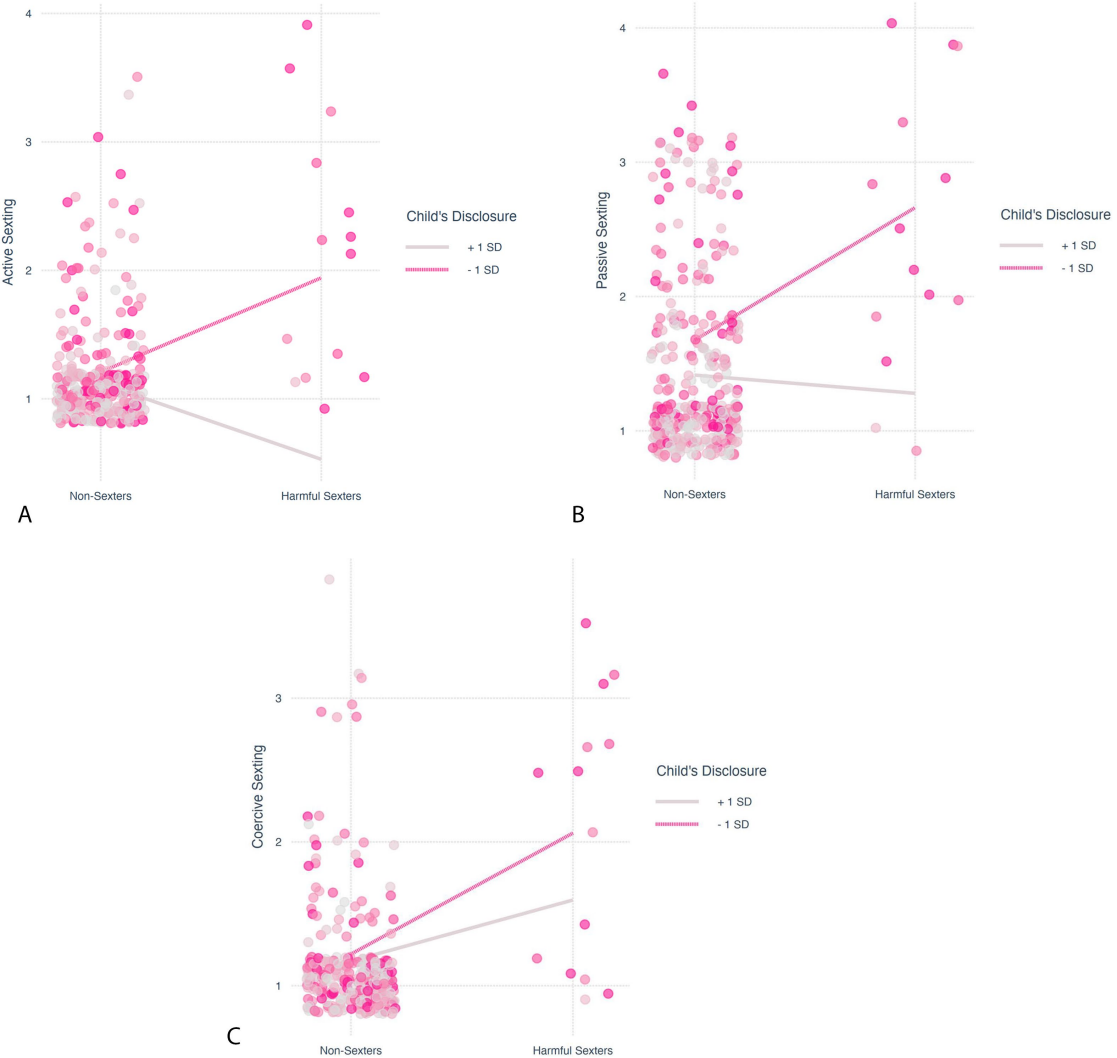
**(a)** Simple slope test to probe the moderating effect of adolescents’ disclosure of information to parents on the association between profiles (non-sexters vs. harmful sexters) and active sexting. **(b)** Simple slope test to probe the moderating effect of adolescents’ disclosure of information to parents on the association between profiles (non-sexters vs. harmful sexters) and passive sexting. **(c)** Simple slope test to probe the moderating effect of adolescents’ disclosure of information to parents on the association between profiles (non-sexters vs. harmful sexters) and coercive sexting.

**Figure 5:**
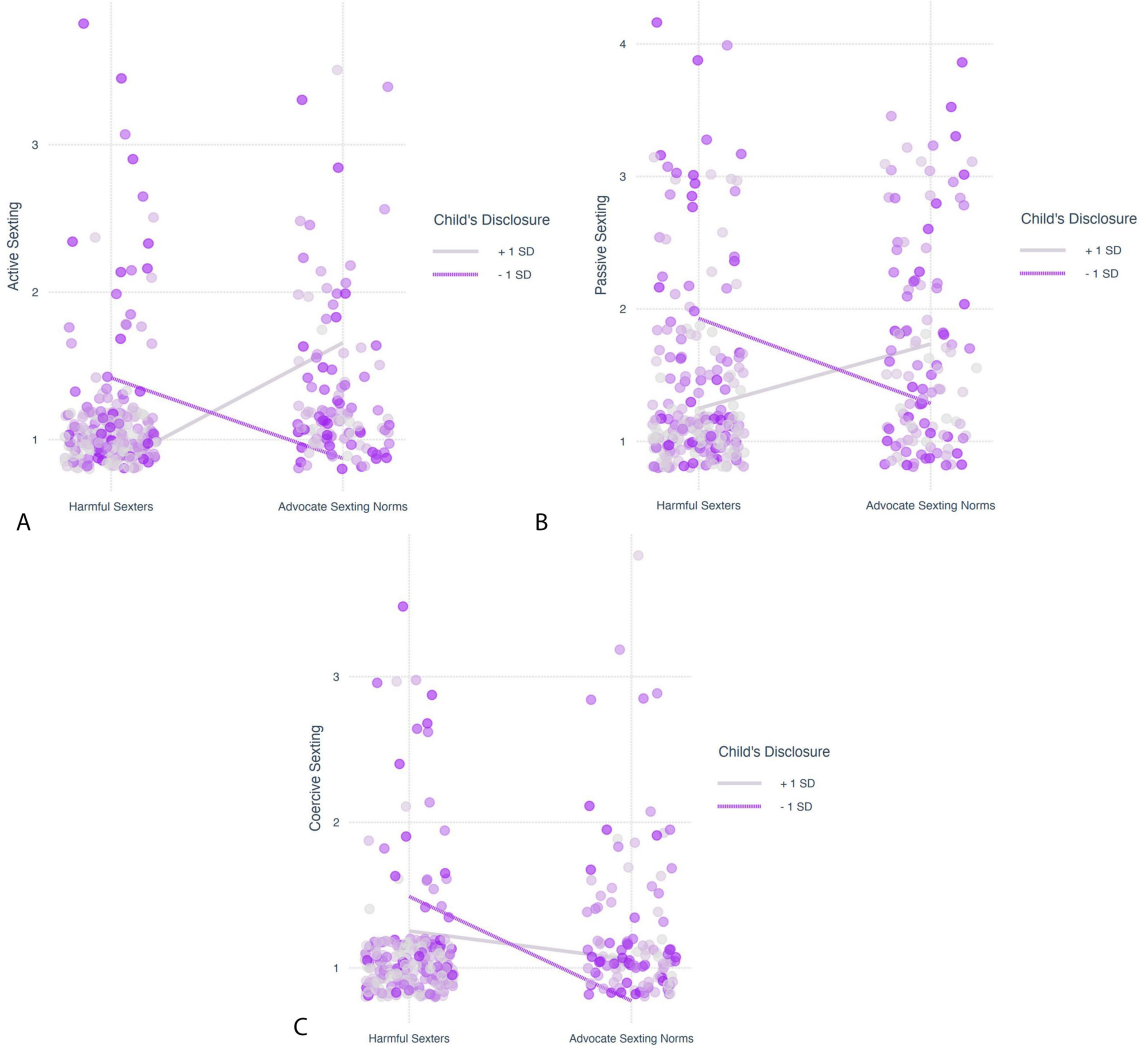
**(a)** Simple slope test to probe the moderating effect of adolescents’ disclosure of information to parents on the association between profiles (advocate sexting norms vs. harmful sexters) and active sexting. **(b)** Simple slope test to probe the moderating effect of adolescents’ disclosure of information to parents on the association between profiles (advocate sexting norms vs. harmful sexters) and passive sexting. **(c)** Simple slope test to probe the moderating effect of adolescents’ disclosure to parents on the association between profiles (advocate sexting norms vs. harmful sexters) and coercive sexting.

The analyses revealed that adolescents’ disclosure of information to parents significantly moderated the effects of the participants’ profiles (harmful sexters vs. non-sexters and harmful sexters vs. advocates of sexting norms) on sexting behaviors. Regression results are presented in Table 3. The correlations between sexting behaviors (Figure 3) revealed only moderate-to-strong associations, suggesting that these moderating effects were not highly dependent. Simple slope effects are presented in Figures 4a–c, 5a–c.

**Table 3:** Regression coefficients for predicting sexting behaviors by profiles and disclosure of information to parents.

	Active sexting			Passive sexting			Coercive sexting		
Predictors	Estimates	CI	*p*	Estimates	CI	*p*	Estimates	CI	*p*
(Intercept)	1.20	1.13–1.27	**<0.001**	1.60	1.50–1.71	**<0.001**	1.23	1.16–1.29	**<0.001**
Harmful vs. Advocating	0.14	−0.24 to 0.53	0.464	−0.08	−0.67 to 0.51	0.795	−0.46	−0.85 to −0.07	**0.021**
Harmful vs. None	-0.07	−0.45 to 0.31	0.717	−0.43	−1.01 to 0.16	0.153	−0.63	−1.02 to −0.25	**0.001**
Disclosure to parents	−0.01	−0.03 to 0.00	0.111	−0.04	−0.06 to −0.02	**<0.001**	−0.00	−0.02 to 0.01	0.521
Gender	0.05	−0.04 to 0.14	0.281	−0.29	−0.43 to −0.16	**<0.001**	−0.11	−0.20 to −0.02	**0.016**
Religiosity (secular vs. traditional)	−0.04	−0.13 to 0.05	0.370	0.08	−0.06 to 0.22	0.262	−0.03	−0.12 to 0.06	0.486
Religiosity (secular vs. religious)	−0.08	−0.29 to 0.12	0.433	−0.20	−0.52 to 0.11	0.211	−0.10	−0.31 to 0.11	0.351
Harmful vs. Advocating × Disclosure	0.22	0.14–0.30	**<0.001**	0.18	0.05–0.30	**0.005**	0.08	0.00–0.16	**0.050**
Harmful vs. None × Disclosure	0.21	0.13–0.29	**<0.001**	0.18	0.05–0.30	**0.005**	0.07	0.01–0.15	**0.049**
Observations	345			345			345		
*R*^2^ / *R*^2^ adjusted	0.290/0.273			0.271/0.254			0.214/0.195		

Note. Bold values indicate statistically significant effects (p < 0.05). Estimates represent regression coefficients, and CI indicates 95% confidence intervals. The reference group for the profile comparisons is the Advocating profile. Gender and religiosity variables were entered as categorical predictors according to the contrasts indicated in the table.

We found a consistent moderating effect: for participants with a high inclination to disclose information to parents (one standard deviation above the sample mean), no differences were found between the profiles in any of the sexting behaviors (harmful sexters vs. non-sexters in active, *b* = 0.59, *SE* = 0.54, *t* = 1.10, *p* = 0.27, passive, *b* = 0.14, *SE* = 0.78, *t* = 0.17, *p* = 0.86, and coercive sexting, *b* = −0.43, *SE* = 0.70, *t* = −0.61, *p* = 0.54; harmful sexters vs. advocates of sexting norms in active, *b* = 0.83, *SE* = 0.54, *t* = 1.54, *p* = 0.12, passive, *b* = 0.49, *SE* = 0.78, *t* = 0.62, *p* = 0.53, and coercive sexting, *b* = −0.20, *SE* = 0.70, *t* = −0.28, *p* = 0.78). Conversely, among participants with a low inclination to disclose information to parents (one standard deviation below the sample mean), the harmful sexters engaged in more sexting behaviors (active, passive, and coercive) than either non-sexters (active, *b* = −0.73, *SE* = 0.22, *t* = −3.29, *p* < 0.01, passive, *b* = −0.99, *SE* = 0.30, *t* = −3.27, *p* < 0.01, and coercive sexting, *b* = −0.84, *SE* = 0.26, *t* = −3.28, *p* < 0.01) or those advocating sexting norms (active, *b* = −0.55, *SE* = 0.23, *t* = −2.41, *p* = 0.02, passive, *b* = −0.64, *SE* = 0.31, *t* = −2.08, *p* = 0.04, and coercive sexting, *b* = −0.72, *SE* = 0.26, *t* = −2.76, *p* = 0.01). These effects were significant after adjusting for the contribution of gender and religiosity.

## 4 Discussion

The present study identified three latent profiles of adolescents based on their attitudes toward sexting, victim-blaming, harm minimization with respect to IBSA and social desirability: non-sexters, advocates of sexting norms, and harmful sexters. The profiles represent meaningful psychological and behavioral distinctions in adolescent engagement with sexting and their associated normative frameworks.

Non-sexters comprised the majority of the sample. They tended to rely more on social norms that do not encourage sexting and displayed higher social desirability, indicating that they adopted norms that oppose sexting and possibly internalized the prevalent message about the dangers of sending nude photos that is disseminated by significant adults like parents and educators (Cuccì et al., 2024). This group also engaged less in victim blaming and harm minimization. It appears that the changing social norms following awareness (Eaton et al., 2024) in the post-MeToo era (Fileborn and Loney-Howes, 2019) are permeating the younger generation, who are less inclined to blame victims and downplay the harm caused to those affected. There also appears to be growing awareness of harm in the online space. These findings align with those of previous studies that identified non-engagement in sexting as reflective of higher levels of family and peer cohesion (Dolev-Cohen, 2024), self-discipline, responsible behavior (Kokkinos et al., 2024), and greater self-regulation (Holfeld et al., 2024).

By contrast, the group of advocates of sexting norms, comprising about one-third of the participants, exhibited greater openness toward sexting as a normative behavior but did not strongly endorse victim-blaming or harm minimization attitudes. They reported below-average social desirability, indicating reduced concern with impression management. This group represents adolescents who perceive sexting as an accepted part of peer relationships but remain within a normative boundary that does not condone harmful practices. The profile describes what researchers referred to as sexting as a normative behavior, where adolescents participate in digital sexual expression in ways that are socially constructed as non-deviant (Holfeld et al., 2024; Van Ouytsel et al., 2018; Dolev-Cohen, 2023).

The smallest group was that of harmful sexters, who presented the highest levels of harm minimization and victim-blaming tendencies, in addition to a full acceptance of sexting as the norm. This group also exhibited low social desirability, suggesting a reduced inclination to conceal socially sensitive behaviors. Their profile is particularly worrisome given their increased likelihood of coercive sexting, as revealed in the moderation analysis. These adolescents may internalize problematic peer norms that legitimize exploitative or aggressive digital sexual conduct. This finding supports those of other studies showing that sexting may be prompted by instrumental motives (Dolev-Cohen, 2023) and exhibit markers of engagement in risk behaviors (Van Ouytsel et al., 2018). From the criminological perspective, this pattern may be explained by Sutherland’s (1947) differential association theory, according to which deviant behavior is learned by interaction with intimate groups that promote definitions favorable to violations of the law. Thus, harmful sexters may be part of peer environments where exploitative sexting is normalized, shaping their attitudes and behaviors accordingly. Indeed, during adolescence, peer group norms are usually considered to be among the most influential factors in adolescents’ sexual development and behaviors (Buhi and Goodson, 2007).

A critical finding of this study is the moderating role of adolescents’ disclosure to their parents. Among adolescents reporting high parental disclosure, profile membership was not associated with differences in active, passive, or coercive sexting behaviors. This suggests that open parent–child communication can serve as a protective buffer, mitigating the behavioral risks associated with deviant attitudes and norms. By contrast, among adolescents with low parental disclosure, the harmful sexters engaged significantly more in all forms of sexting than in either of the other groups. Such an interaction effect illustrates the pivotal role of the child–parent relationship in adolescent digital behavior. These findings echo previous research indicating that open communication with parents serves as a form of informal social control, restraining risky behaviors (Kerr and Stattin, 2000; Sher-Censor et al., 2021). Regarding the online space, studies found that beneficial communication between parents and their adolescent children was important for mediating online behavior and quality discourse (Boniel-Nissim et al., 2020; Cuccì et al., 2024; Dolev-Cohen and Ricon, 2022). According to social control theory (Hirschi, 1969), the adolescents’ open communication with their parents creates commitment to social norms and values accepted by the parents, producing a balancing alternative position that reduces the negative influences of the environment. The moderating pattern reinforces the dual-process model of adolescent development, where risky attitudes lead to behavior only when unbuffered by parental guidance. It also complements psychological models of adolescent secrecy, suggesting that low disclosure not only limits parental knowledge but may also signal relational detachment that makes deviance possible (Tilton-Weaver, 2014).

Importantly, the moderating role of parental disclosure reflects adolescents’ recollections of parent–child communication during high school, rather than current parental involvement among young adults.

In sum, the findings demonstrate the importance of differentiating adolescents not only by their sexting behaviors but also by their attitudinal profiles and family dynamics. Some adolescents hold normative views on sexting without engaging in harmful behavior but others internalize attitudes that may lead to digital aggression. These risks are not unavoidable, however. Parental engagement, specifically, leading to adolescents’ willingness to share personal experiences, emerges as a powerful moderator that can neutralize the behavioral expression of risky profiles.

### 4.1 Limitations and potentialities

The present study has several limitations. First, the reliability of the social desirability questionnaire was found to be low, and the research findings should be interpreted accordingly. Additionally, the harmful sexters’ group was very small, which may limit the statistical power and generalizability of the findings regarding this group, requiring additional studies to strengthen the findings. In addition, although the overall sample size approaches common recommendations for LPA, the smallest profile was relatively small. While person-centered analyses are robust to unequal class sizes, replication with larger samples is warranted. Finally, although the sample includes participants aged 16–26, all measures referred explicitly to experiences during adolescence in high school. Nevertheless, retrospective reporting may be subject to recall bias, and future studies would benefit from longitudinal designs following adolescents in real time. Despite these limitations, the study offers several methodological and conceptual strengths. First, it adopts a theory-driven person-centered approach that captures heterogeneity in adolescents’ sexting-related attitudes rather than relying on variable-centered associations alone. Second, by integrating normative constructs such as victim blaming and harm minimization with family communication processes within a single analytical model, it advances a more ecologically grounded understanding of adolescent sexting as a socially embedded developmental phenomenon. Finally, combining latent profile analysis with a moderation framework moves beyond identifying risk configurations to clarifying the relational conditions under which such configurations become behaviorally consequential. Together, these contributions strengthen the theoretical precision and practical relevance of the findings.

### 4.2 Conclusion and implications

The present findings have several implications for educators, parents, and policymakers aiming to promote adolescent wellbeing in the digital realm. From a developmental-criminological perspective, the three sexting profiles revealed by the study illustrate how theory and practice converge on the protective role of parent–child communication. According to social control theory, strong bonds to family, like warm attachment and open disclosure of information to parents, tether youths to pro-social norms and reduce deviance (Pistoni et al., 2023). By contrast, differential association theory suggests that adolescents learn and normalize behaviors like sexting through peer influences and exposure to attitudes that favor such conduct (Walrave et al., 2015; Confalonieri et al., 2020). The finding that adolescents’ disclosure of information to parents moderates sexting behavior aligns with these models. When parent–child communication is high, even adolescents inclined toward sexting, including those with a harmful sexter profile, show risk levels comparable to non-sexters, an indication that parental involvement serves as a buffer against peer-driven sexting risks. This pattern reflects social control in action: open disclosure and trust serve as informal social control, anchoring adolescents to family expectations and deterring online sexual deviance (Cioban et al., 2021). By contrast, when disclosure is low, parental influence wanes and differential association processes dominate. Youths immersed in peer environments that normalize risky sexting are far more likely to engage in harmful sexting practices, which explains why harmful sexters exhibited significantly greater risk under weak parent communication. These insights have important practical implications, suggesting that parents should prioritize building trust and dialogue around technology and sexuality because adolescents who feel safe disclosing their online experiences tend to explore digital sexuality more cautiously and responsibly (Towner et al., 2023). Rather than relying on strict surveillance or punitive measures, a collaborative approach is advised, educators and policymakers helping families establish and reinforce healthy open communication norms and accountability both at home and in the classroom (Dolev-Cohen, 2023; Del Rey et al., 2021), for example, through school programs that encourage parent-adolescent discussions about sexting and online conduct. Parenting strategies based on social control and social learning principles, together with educational policies that promote parent-school alliances, can leverage the proven protective effect of parental disclosure to mitigate sexting-related risks and foster safer online behaviors of adolescents.

These implications are relevant primarily to adolescence, as the reported behaviors and family dynamics refer to the high school period.

## Data Availability

The raw data supporting the conclusions of this article will be made available by the authors, without undue reservation, upon reasonable request.
